# Inconvenience of Living Place Affects Individual HbA1c Level in a Rural Area in Japan: Shimane CoHRE Study

**DOI:** 10.3390/ijerph18031147

**Published:** 2021-01-28

**Authors:** Rie Fukuoka, Miwako Takeda, Takafumi Abe, Masayuki Yamasaki, Shinji Kimura, Kenta Okuyama, Minoru Isomura, Toru Nabika

**Affiliations:** 1 Department of Community Health and Gerontological Nursing, Faculty of Medicine, Shimane University, Izumo, Shimane 693-8501, Japan; 2 Department of Functional Pathology, Faculty of Medicine, Shimane University, Izumo, Shimane 693-8501, Japan; nabika@med.shimane-u.ac.jp; 3 The Center for Community-Based Healthcare Research and Education (CoHRE), Shimane University, Izumo, Shimane 693-8501, Japan; cohre1@med.shimane-u.ac.jp (M.T.); t-abe@med.shimane-u.ac.jp (T.A.); myamasak@hmn.shimane-u.ac.jp (M.Y.); s-kimura@med.shimane-u.ac.jp (S.K.); ko215ok@gmail.com (K.O.); isomura@hmn.shimane-u.ac.jp (M.I.); 4 Faculty of Human Sciences, Shimane University, Matsue, Shimane 690-8504, Japan; 5 Department of Clinical Nursing, Faculty of Medicine, Shimane University, Izumo, Shimane 693-8501, Japan; 6 Center for Primary Health Care Research, Lund University, Jan Waldenströms gata 35, 20502 Malmö, Sweden

**Keywords:** diabetes mellitus, geographical information system, altitude, a cross-sectional study

## Abstract

Background: It has been shown that the socio-geographical environment of residential areas, such as altitude, affects the health status and health-maintenance behavior of residents. Here, we examined a hypothesis that altitude of residence would influence glycemic control in a general elderly population living in a rural area. Methods: A thousand and sixteen participants living in a mountainous region in Japan were recruited at health examinations. Hemoglobin A1c (HbA1c) was measured in serum as a parameter of glycemic control. The altitude of residence, distance to grocery stores and to medical facilities were estimated using a geographic information system. Results: Linear regression analysis confirmed a significant effect of the altitude on log HbA1c even after adjustment of other demographic and biochemical factors. When the distance to grocery stores or medical facilities were used instead of the altitude in a linear regression analysis, distance to secondary medical facilities alone showed a significant effect on HbA1c. Conclusions: We found a positive correlation between HbA1c level and residential altitude in a rural area of Japan. The altitude seemed to be a parameter substituting the inconvenicence of residential areas. Socio-geographical factors of living place, such as inconvenience, may influence glycemic control of the residents.

## 1. Introduction

The number of diabetic patients has been increasing rapidly due to changes in lifestyle and social circumstances in Japan as well as in the world [1,2]. In addition to causing severe complications such as retinopathy, neuropathy and nephropathy, diabetes is a major risk for cardiovascular diseases such as stroke and ischemic heart disease, which are all serious burdens not only for patients and their families but also for society [3,4,5].

For effective prevention of diabetes, it is essential to clarify as many risk factors as possible. In terms of risk factors for diabetes and obesity, intense studies have been performed on genetic factors, individual lifestyle such as exercise and eating habits [6]. In addition to such classical risk factors, socio-geographical factors have been focused as a potential risk factor for obesity and/or diabetes [7]; previous studies showed that residents in an area with “health-harming” food outlets had higher odds of developing diabetes and obesity [8,9], and a higher level of walkability was associated with a lower risk of diabetes [10]. These socio-geographical factors were thought to influence the risk of diabetes through modification of individual behavior (e.g., exercise and eating). Further, greater altitude was reported to improve insulin resistance and reduce the risk of diabetes, possibly through changes in glycemic metabolisms in the body [11,12,13].

Recently, we showed that the altitude of the residential area affected salt intake and hypertension in a rural area [14,15]. In these studies, subjects living in higher places showed greater salt intake and prevalence of hypertension. Based on these observations and previous studies, we hypothesized that altitude, one of the basic geographical features of a residential area, could affect glycemic control of individuals in a rural area, which may influence the risk of diabetes.

To examine the hypothesis above, the objective of this study was to clarify the relationship between the altitude of a residential area and individual Hemoglobin A1c (HbA1c). We employed HbA1c since it was a good parameter for long-term glycemic control.

## 2. Materials and Methods

### 2.1. Participants

**Figure 1:**
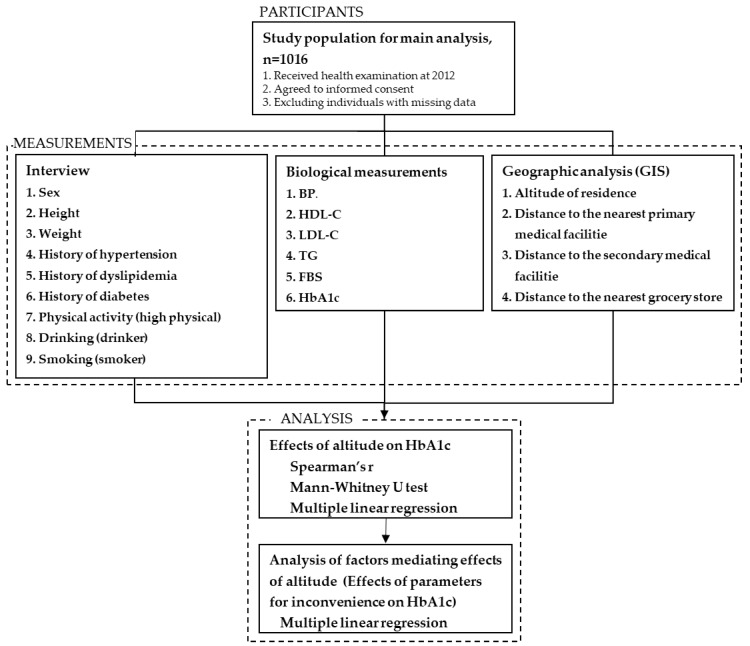
Materials and methods. Abbreviations; BP—blood pressure, HDL-C—high-density lipoprotein cholesterol, LDL-C—low-density lipoprotein cholesterol, TG—triglyceride, FBS—fasting blood glucose, HbA1c—hemoglobin A1c.

We performed a cross-sectional study recruiting residents of a rural mountainous area in Japan to assess the association of residential altitude as well as parameters of inconvenience with HbA1c (Figure 1). This cross-sectional population-based study was conducted as a part of the Shimane CoHRE Study, a cohort study designed to determine risk factors of lifestyle-related diseases [16,17,18,19,20,21,22,23,24]. Health examination was performed in Un-nan City in 2012. Un-nan City is located in a rural mountainous area in the eastern part of Shimane Prefecture, Japan. People receiving health examination were invited to participate in the study and were included when they gave written informed consent. After excluding individuals with missing data, we recruited 1016 individuals in the study. The protocol of this study was approved by the ethics committee of Shimane University (#2888). Written informed consent was obtained from all participants.

### 2.2. Measurements

Interviews were completed in a health examination regarding the history of hypertension, dyslipidemia and diabetes. Medications for these diseases were confirmed by reviewing prescription records. Regular physical activity, alcohol consumption and smoking habit were obtained in the interview. Subjects who routinely took 1 h or more of physical activity (for example, walking) per day were categorized as those with high physical activity. Drinkers were defined as those who drank alcohol regularly (more than once a week). Smokers were defined as those (i) who had ever smoked either more than 100 cigarettes or for more than 6 months, and (ii) who had smoked within the last one month. Drivers were defined as those who had a driving license and drove regularly. Blood pressure (BP) was measured twice at the site with automatic sphygmomanometers after a 15-min of rest in the sitting position, and the lower value was taken as the representative BP.

Serum was taken from the participants after at least 6-h fasting, and high-density and low-density lipoprotein cholesterol (HDL-C and LDL-C, respectively), triglycerides (TG), fasting blood glucose (FBS), and HbA1c were measured by standard methods.

The altitude of residence was estimated with a geographic information system (GIS) based on the address of participants (ESRI Japan, Tokyo, Japan). In the analysis, subjects were divided into quartile groups according to altitude of their residence; 0–44 m (N = 262), 45–68 m (N = 247), 69–195 m (N = 250) and 196–485 m (N = 257). The distance to the nearest primary (clinics) and secondary medical facilities (middle-sized hospitals) and to the nearest grocery stores was calculated using a GIS. These distances were not estimated as a straight-line distance but as the shortest distance along with real roads on a map. Although the distance to medical facilities was available for all participants, the distance to grocery stores was not available in some areas. Accordingly, the data on grocery stores for 658 participants were used in the following analysis.

### 2.3. Statistical Analysis

The measures were represented as the mean and the 95% confidence interval. Log-transformed HbA1c and TG were used in the analyses due to their skewed distribution. For univariate analyses, ANOVA, the contingency table analysis and the nonparametric Mann–Whitney U test and Spearman’s rank method were employed when appropriate. Parameters influencing HbA1c were then analyzed by linear regression analysis. *p* < 0.05 was considered statistically significant. All of the statistical analyses were performed using SPSS (v.22, IBM, Armonk, NY, USA).

## 3. Results

Demographic data of the studied population are shown in Table 1. HbA1c level differed significantly among the quartiles of altitude, and further, it showed a significant linear trend along with the quartiles of altitude. In addition, the altitude had significant effects on the age of residents, although the linear trend with the altitude was not significant.

Factors correlating with log HbA1c are shown in Table 2. BMI, HDL-C and log TG were found to have significant correlations with HbA1c in addition to the altitude of residence.

Based on this univariate correlation analysis, we then performed a linear regression analysis on the effects of those parameters on HbA1c. The result is summarized in Table 3A. The altitude (in quartiles) showed an independent influence on HbA1c in addition to BMI and TG. Co-linearity among the parameters was not high when variance inflation factors (VIFs) were calculated (the maximal VIF for the altitude was 1.48). The significant influence of the altitude on HbA1c was confirmed in the analysis in which the altitude was input as a real value (results not shown).

As inconvenience may explain the effect of the altitude on HbA1c, correlation of the altitude and distance to the nearest medical facilities (primary or secondary medical facilities) as well as to the nearest grocery stores was examined in Table S1. The result indicated that distance to medical facilities was positively correlated with the quartile of altitude and with the real value of altitude. In particular, distance to secondary medical facilities showed a high correlation with altitude. In contrast, distance to grocery stores was negatively correlated with the quartile of altitude (see Table S1). To examine whether these parameters for inconvenience underlying the altitude mediated effects of altitude, linear regression analyses were performed in which those parameters were substituted for the altitude. As a result, only the distance to secondary medical facilities showed a significant association with HbA1c (Table 3B–D).

## 4. Discussion

In this study, our objective was to examine the effects of residential altitude on the HbA1c level of the residents, and we actually found a positive correlation of the altitude with HbA1c even under consideration of other confounding factors. This was shown in both two linear regression models using the altitude either in real values or in the form of a categorized variable. Further, when a standardized *β* was taken into account, the effect size of altitude was as large as that of BMI, while HDL-C and TG had only a marginal effect. In another word, the altitude had as a robust effect as a classical risk factor, BMI, in this studied population.

There are various hypotheses on mechanisms of how altitude affects health conditions; the influence of altitude on health may be mediated by oxygen concentration, climate, including temperature, and physical burden caused by slopes [25,26]. This was also in the case in diabetic status or glycemic control; Schobersberger et al. reported that, after staying 3 weeks at an altitude of 1700 m, insulin resistance estimated by HOMA-IR was improved [11]. In addition, two more reports indicated that 3 days of a mountain hike at an altitude of 2400 m significantly improved glucose tolerance of sedentary subjects [12], and a condition simulating that of 4300 m above the sea level reduced serum insulin concentrations after glucose loading significantly [13]. However, it was not likely that climate and oxygen concentration (thin air) had a significant influence on diabetic status in our population because the altitudes of residence were less than 500 m.

On the other hand, it was possible that slopes around residential areas at a high altitude hampered physical activity and worsened diabetic status [27,28]. This was more likely if the age of the population studied here was considered. However, the observation that physical activity per se had no significant effects on HbA1c did not support this hypothesis (see Table 2). Daily use of cars may reduce physical activity in any way, which may erase potential effects of altitude (or slope) on physical activity [29]. A detailed analysis would be required on this issue in future studies.

Inconvenience is another key concept when the influence of altitude on health is considered. In another word, inconvenience underlying high altitude may be a major mediator of the influence of altitude. In the present study, distance not to primary but to secondary medical facilities was found to be associated with HbA1c level. This was an interesting observation since the distance to primary and secondary medical facilities was increased significantly along with the altitude (see Table S1). Takeda et al. found that diabetic patients living in this region were more likely to choose medical facilities in a distant urban area. Takeda et al. speculated that diabetic patients preferred doctors specialized in diabetes who were working in larger hospitals in an urban area [30]. This consultation behavior may contribute to the observation above (i.e., distance not to primary, but to secondary medical facilities associated with the status of glycemic control). A similar discrepancy between the effects of primary and secondary medical facilities on a disease condition was observed in poorly controlled hypertension [31]. For better chronic care of lifestyle diseases such as hypertension and diabetes, primary and secondary medical facilities may need to divide their tasks according to their roles in the regions [32]. On the other hand, there is another possibility that distance to secondary medical facilities may be a substitute for still-unknown parameters of inconvenience. As secondary medical facilities are expected to be located in urban areas, facilities other than hospitals in such urban areas may be a true contributor.

The distance to grocery stores did not affect the HbA1c level in the linear regression model. This observation suggested that closeness to grocery stores did not promote overconsumption of food in our population. This is consistent with a previous observation that each mile closer to a fast food restaurant was associated with a lower prevalence of obesity [9]. Distance to food shops and/or restaurants may not necessarily influence food consumption and body weight of residents.

The altitude may have additional effects on HbA1c through pathways other than the inconvenience (or distance to medical facilities and grocery stores). To test this possibility, a model including all the parameters for the inconvenience and the altitude was examined. This model, however, did not improve the fitness of the model and, further, caused a distorted result, i.e., the effect of BMI on HbA1c, which was robust in other models, was disappeared (result not shown). This might be due to substantial correlations of the parameters with one another (see Table S1).

We have several limitations in this study; first, distance to grocery stores was missing in some areas. As the distance to grocery stores was previously reported to affect health conditions [33,34,35], a close examination of its effects on diabetic status in a future study. Second, as this study focused on elderlies in one mountainous region of Shimane Prefecture, the result should be confirmed in studies on middle-aged populations as well as in other regions. Third, this was a cross-sectional study that was not suitable to infer a causal relationship. In spite of that, residential altitude was not likely to be determined by individual HbA1c, and thus it was feasible to assume that the altitude had some causal effects on individual HbA1c probably through other factors such as socio-geographical inconvenience. Finally, although our result supported our hypothesis that altitude of living place affected the risk of diabetes, no direct effect of the altitude on the incidence of diabetes per se was not explored in this study, longitudinal studies to evaluate the effect of the altitude on the incidence of diabetes would be essential.

## 5. Conclusions

HbA1c was significantly associated with the altitude of residence even after adjustment of other potential factors. Distance to medical facilities, especially to secondary medical facilities, was suggested to be an important factor mediating the influence of the altitude.

## Figures and Tables

**Table 1:** Demographic data of the studied subjects.

	Quartile 1	Quartile 2	Quartile 3	Quartile 4	*p*	Effect Size
Number	262	247	250	257		
Altitude, m	38 (38, 39)	53 (52, 54)	123 (118, 128)	304 (294, 314)	*p* < 0.001	-
Age, years	71 (70, 72)	70 * vs. ^4^ (69, 71)	70 (69, 71)	71 (70, 72)	0.01	0.011 ^b^
Sex, % male	37.4	38.1	41.6	41.6	0.65	0.04 ^c^
BMI, kg/m^2^	22.2 (21.8, 22.6)	22.2 (21.8, 22.7)	21.9 (21.5, 22.2)	22.5 (22.2, 22.9)	0.09	0.006 ^b^
SBP, mmHg	129 (127, 131)	128 (126, 130)	127 (125, 129)	128 (126, 130)	0.78	0.001 ^b^
DBP, mmHg	77 (76, 79)	78 (76, 79)	76 (75, 78)	77 (76, 79)	0.49	0.002 ^b^
Antihypertensive drug, % yes	35.9	32.8	38.4	43.6	0.08	0.081 ^c^
HDL-C, mg/dL	66 (64, 68)	64 (62, 66)	66 (64, 68)	63 (61, 65)	0.14	0.005 ^b^
LDL-C, mg/dL	123 (119, 126)	124 (120, 127)	118 (115, 122)	121 (118, 125)	0.17	0.005 ^b^
TG ^a^, mg/dL	85.4 (81.3, 89.7)	89.1 (84.3, 94.2)	83.7 (79.5, 88.1)	83.4 (79.3, 87.8)	0.26	0.004 ^b^
Antihyperlipidemic drug, % yes	23.3	17.8	18.8	21.4	0.41	0.054 ^c^
HbA1c ^a^, %	5.32 (5.27, 5.37)	5.35 (5.30, 5.41)	5.39 (5.33, 5.44)	5.48 * vs. ^1, 2^ (5.43, 5.54)	*p* < 0.001	0.019 ^b^
Antidiabetic drugs, % yes	4.6	3.6	5.6	5.4	0.72	0.036 ^c^
Activity, % high activity	47.3	46.6	48.0	50.6	0.82	0.03 ^c^
Drinking, % yes	44.7	42.3	54.0	47.7	0.05	0.087 ^c^
Smoking, % yes	5.3	6.5	4.0	5.4	0.67	0.039 ^c^
Driving, % yes	67.9	72.0	76.8	67.3	0.07	0.083 ^c^

Abbreviations; BMI—body mass index, SBP and DBP—systolic and diastolic blood pressure, HDL-C—high-density lipoprotein cholesterol, LDL-C—low-density lipoprotein cholesterol, TG—triglyceride, HbA1c—hemoglobin A1c. ^a^ log-transformed HbA1c and TG were used in the analysis, and a 95% confidence interval is shown in a blanket instead of SD. *; significantly different from the quartile 4 (vs. ^4^) or the quartiles 1 and 2 (vs. ^1^, ^2^). Bonferroni’s test was used for multiple comparisons. ^b^, ^c^; to obtain effect sizes, η^2 b^ or Cramer’s V ^c^ was used.

**Table 2:** Factors correlated with log HbA1c.

	*ρ* or Z *	*p*
Age	−0.02	0.55
Sex, M vs. F	−1.00	0.32
BMI	0.10	<0.001
SBP	0.00	0.99
DBP	−0.03	0.38
HDL-C	−0.15	<0.001
LDL-C	0.05	0.10
log TG	0.11	<0.001
Smoking, Y vs. N	−0.50	0.62
Drinking, Y vs. N	−1.40	0.16
High physical activity, Y vs. N	−0.68	0.50
Driving, Y vs. N	−0.19	0.85
Altitude	0.16	<0.001

Abbreviations; HbA1c – hemoglobin A1c, BMI—body mass index, SBP and DBP—systolic and diastolic blood pressure, HDL-C—high-density lipoprotein cholesterol, LDL-C—low-density lipoprotein cholesterol, TG—triglyceride, M—male, F—female, Y—yes, N—no. *; *ρ* and *Z* are shown for Spearman’s rank method and Mann–Whitney U test, respectively.

**Table 3:** Linear regression analysis on log HbA1c.

(**A**) A model including altitude					
	**B**	**SE**	**Standardized**	**t**	***p***
			***β***		
Age, per 10 years	0.0001	0.002	0.002	0.07	0.94
Sex, M vs. F	0.002	0.002	0.03	0.81	0.42
BMI	0.001	0.0004	0.11	3.38	0.001
HDL-C	−0.0001	0.0001	−0.07	−1.80	0.07
log TG	0.014	0.007	0.07	1.98	0.05
Altitude, quartile	0.004	0.001	0.13	4.23	<0.001
(**B**) A model including distance to primary medical facilities					
	**B**	**SE**	**Standardized**	**t**	***p***
			***β***		
Age, per 10 years	0.0001	0.002	0.003	0.10	0.92
Sex, M vs. F	0.002	0.002	0.03	0.79	0.43
BMI	0.001	0.0004	0.12	3.49	0.001
HDL-C	−0.0002	0.0001	−0.07	−1.99	0.05
log TG	0.012	0.007	0.06	1.68	0.09
Distance to the nearest primary medical facility	0.0003	0.001	0.02	0.49	0.63
(**C**) A model including distance to secondary medical facilities					
	**B**	**SE**	**Standardized**	**t**	***p***
			***β***		
Age, per 10 years	−0.0002	0.002	−0.004	−0.14	0.89
Sex, M vs. F	0.002	0.002	0.02	0.79	0.43
BMI	0.001	0.0004	0.11	3.17	0.002
HDL-C	−0.0002	0.0001	−0.07	−1.94	0.05
log TG	0.014	0.007	0.07	1.99	0.05
Distance to the nearest secondary medical facility	0.001	0.0002	0.17	5.70	<0.001
(**D**) A model including distance to grocery stores					
	**B**	**SE**	**Standardized**	**t**	***p***
			***β***		
Age, per 10 years	−0.0004	0.002	−0.01	−0.22	0.83
Sex, M vs. F	0.003	0.003	0.04	0.97	0.33
BMI	0.001	0.0005	0.08	1.97	0.05
HDL-C	−0.0002	0.0001	−0.11	−2.45	0.01
log TG	0.009	0.008	0.05	1.06	0.29
Distance to the nearest grocery store	−0.0002	0.0004	−0.02	−0.61	0.54

Abbreviations: BMI—body mass index, HDL-C—high-density lipoprotein cholesterol, TG—triglyceride.

## Data Availability

Data cannot be shared publicly because of ethical issues. Data will be available from the CoHRE under permission of the Ethics Committee of Shimane University. Anyone who wants to access the data should contact with the corresponding author.

## Supplementary Materials

The following are available online at https://www.mdpi.com/1660-4601/18/3/1147/s1, Table S1: (A) Distance between the residence and the nearest medical facility/grocery store, (B) Correlation between the altitude and the distance to medical facility/grocery store.
